# Assessment of Bitterness in Non-Charged Pharmaceuticals with a Taste Sensor: A Study on Substances with Xanthine Scaffold and Allopurinol

**DOI:** 10.3390/molecules29112452

**Published:** 2024-05-23

**Authors:** Zeyu Zhao, Fang Song, Shunsuke Kimura, Takeshi Onodera, Takahiro Uchida, Kiyoshi Toko

**Affiliations:** 1 Graduate School of Information Science and Electrical Engineering, Kyushu University, 744 Motooka, Nishi-ku, Fukuoka 819-0395, Japanonodera@ed.kyushu-u.ac.jp (T.O.); 2 Research and Development Center for Five-Sense Devices, Kyushu University, 744 Motooka, Nishi-ku, Fukuoka 819-0395, Japan; 3 Faculty of Nutritional Sciences, Nakamura Gakuen University, 5-7-1 Befu, Jonan-ku, Fukuoka 814-0198, Japan; 4 Food and Health Innovation Center, Nakamura Gakuen University, 5-7-1 Befu, Jonan-ku, Fukuoka 814-0198, Japan; 5 Faculty of Pharmaceutical Science, Mukogawa Women’s University, 11-68 Koshien 9-Bancho, Nishimiya 663-8179, Japan; 6 Institute for Advanced Study, Kyushu University, 744 Motooka, Nishi-ku, Fukuoka 819-0395, Japan; 7 Graduate School of Nutritional Sciences, Nakamura Gakuen University, 5-7-1 Befu, Jonan-ku, Fukuoka 814-0198, Japan

**Keywords:** taste sensor, allostery, xanthine derivatives, allopurinol, surface modification

## Abstract

Taste sensors with an allostery approach have been studied to detect non-charged bitter substances, such as xanthine derivatives, used in foods (e.g., caffeine) or pharmaceuticals (e.g., etofylline). In this study, the authors modified a taste sensor with 3-bromo-2,6-dihydroxybenzoic acid and used it in conjunction with sensory tests to assess the bitterness of non-charged pharmaceuticals with xanthine scaffolds (i.e., acefylline and doxofylline), as well as allopurinol, an analogue of hypoxanthine. The results show that the sensor was able to differentiate between different levels of sample bitterness. For instance, when assessing a 30 mM sample solution, the sensor response to acefylline was 34.24 mV, which corresponded to the highest level of bitterness (τ = 3.50), while the response to allopurinol was lowest at 2.72 mV, corresponding to relatively weaker bitterness (τ = 0.50). Additionally, this study extended the application of the sensor to detect pentoxifylline, an active pharmaceutical ingredient in pediatric medicines. These results underscore the taste sensor’s value as an additional tool for early-stage assessment and prediction of bitterness in non-charged pharmaceuticals.

## 1. Introduction

The bitterness of active pharmaceutical ingredients (APIs) is a critical factor in pharmaceutical formulation development, as it can influence patient acceptability and adherence, particularly in pediatric medicine [1]. Bitter tastes can cause discomfort in the mouths or throats of pediatric patients, potentially leading to difficulty swallowing, hesitancy in taking the medication, or even vomiting [2,3,4]. Assessing the sensory acceptability of an API in the early stages of drug discovery and development could significantly enhance the process of API selection [5,6]. In the case of human taste panel tests, ethical and safety concerns limit their use, particularly with drugs lacking toxicity data [7,8]. Additionally, taste perception varies between children and adults, necessitating alternative methods for taste evaluation in pediatric drugs [9,10,11].

In the field of pharmaceutical applications, taste-sensing systems (electronic tongues) have shown promise [12,13,14]. These systems encompass various types of sensors, including electrochemical (voltametric, potentiometric) [15,16,17], optical [18,19], or enzymatic sensors (biosensors) [20,21]. Taste sensors, developed by Toko and co-workers, are electronic tongues that use lipid/polymer membranes as receptors to detect taste substances [22]. The sensors are designed to distinguish and quantify the five basic tastes and astringency by detecting the alteration in membrane potential induced by taste substances [23]. They have also effectively generated sensor outputs that align well with the outcomes of sensory tests carried out by panelists. For instance, Haraguchi et al. demonstrated that a taste sensor designed for drug bitterness exhibited high sensitivity to bitterness and showed a significant correlation with human taste receptor hT_2_R_14_ [24].

Commercialized taste sensing systems, such as SA402B and TS-5000Z (Intelligent Sensor Technology Inc., Atsugi-shi, Kanagawa, Japan), have been widely used in evaluating various tastes, including those of beer [25] and tea [26]. Some commercialized sensors are employed to assess the bitterness of medicines [27], such as the C00 sensor for acidic bitter substances like diclofenac sodium [28], and the BT0 sensor for bitter hydrochloride salts, including quinine hydrochloride and diphenhydramine hydrochloride [29]. However, specific taste sensors like BT0 and C00, designed for bitterness, have limited sensitivity to non-charged bitter substances. This limitation arises from their reliance on potentiometric determination; even if these non-charged bitter substances were adsorbed onto the membrane, they would not effectively alter the charge density at the membrane surface, thus failing to induce a significant change in membrane potential. Therefore, a novel sensing approach was needed using lipid/polymer membranes for non-charged bitter substances.

Our group recently developed a novel taste sensor equipped with an innovative measurement mechanism specifically designed for detecting non-charged bitter substances such as caffeine [30]. This method utilizes 2,6-dihydroxybenzoic acid (2,6-DHBA) as a membrane-modifying agent, enhancing the sensitivity of the modified sensor to caffeine, theophylline, and theobromine. When the 2,6-DHBA-modified sensor is immersed in a caffeine solution, the hydroxyl groups of 2,6-DHBA interact with caffeine, influencing the dissociation of H^+^ on the carboxyl group of 2,6-DHBA. This influence on H^+^ dissociation subsequently leads to an increase in the surface charge density of the membrane, resulting in a change in the membrane potential. The mechanism underlying caffeine detection was identified as allosteric using NMR measurements [31].

Allostery, common in enzymes and receptors, involves ligand binding at an allosteric site that can influence substrate binding at an active site [32,33,34,35,36,37,38]. One study [39] proposed that this mechanism underlies caffeine detection by taste sensors, with the hydroxyl group of 2,6-DHBA serving as the allosteric site and the carboxyl group as the active site. Based on these understandings, our group explored additional modifiers to enhance the sensor’s sensitivity to caffeine, such as 3-bromo-2,6-dihydroxybenzoic acid (3-Br-2,6-DHBA) [39]. Utilizing the sensor modified with 3-Br-2,6-DHBA, the authors detected xanthine derivatives used in pharmaceuticals, including etofylline [40].

Xanthine derivatives, known for their diverse biological activities, are widely utilized in the food and pharmaceutical industries [41,42]. They represent a significant class of therapeutic agents with diverse biological activities, including central nervous system stimulation, anti-inflammation, adenosine receptor antagonism, and antitumor activity [43,44,45,46]. The scaffold of xanthine, with its structural rigidity and versatility, offers significant potential for molecular diversity when creating xanthine derivatives in combinatorial chemistry [47,48]. Our previous research [40] identified three APIs with xanthine scaffolds, etofylline, proxyphylline, and diprophylline, prompting further investigation into other non-charged pharmaceuticals, particularly acefylline and doxofylline, which are known for their bronchodilation effects [49,50]. This study also involves the examination of two additional non-charged pharmaceuticals possessing extra pharmacological properties: pentoxifylline, with potential benefits in preterm neonates with necrotizing enterocolitis [51], and allopurinol, used in the treatment of hyperuricemia [52].

This investigation aims to evaluate the bitterness of non-charged pharmaceuticals, including substances with a xanthine scaffold, such as acefylline and doxofylline, as well as allopurinol. Concentration-dependence assessments were conducted using 3-Br-2,6-DHBA-treated taste sensors and sensory tests for these substances. Additionally, the taste sensor was utilized to assess the bitterness of pentoxifylline. The results indicated that taste sensors equipped with an allostery-based detection mechanism can effectively measure the bitterness of non-charged pharmaceuticals.

## 2. Results and Discussion

### 2.1. Confirmation of Sensitivity for BT0 Sensor and 3-Br-2,6-DHBA-Treated Sensor to Non-Charged Bitter Substances

**Figure 1:**
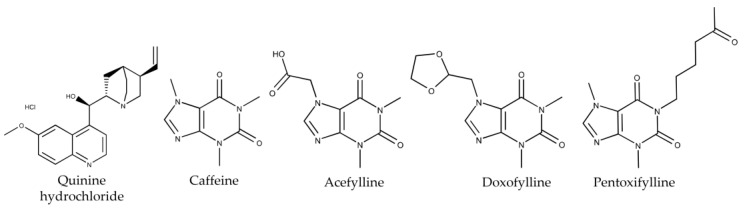
Structural formulas of quinine hydrochloride, caffeine, acefylline, doxofylline, and pentoxifylline.

BT0 sensors, which are commercially available sensors, are capable of detecting the bitter taste of medicines like quinine hydrochloride [23]. The authors investigated the sensitivity of the BT0 sensor to non-charged bitter substances, including caffeine, acefylline, doxofylline, and pentoxifylline. The authors also utilized a taste sensor equipped with lipid/polymer membranes modified by 3-Br-2,6-DHBA to detect these substances. The structural formulas of quinine hydrochloride, caffeine, acefylline, doxofylline, and pentoxifylline are shown in Figure 1. They were dissolved in a reference solution consisting of 30 mM KCl and 0.3 mM tartaric acid, respectively. The measured pH of this reference solution was approximately 3.5, which aligned with the description provided in the previous study [39]. Quinine hydrochloride carries a positive charge upon dissociation in acidic environments, and is referred to hereon as the bitterness (+) sample.

**Figure 2:**
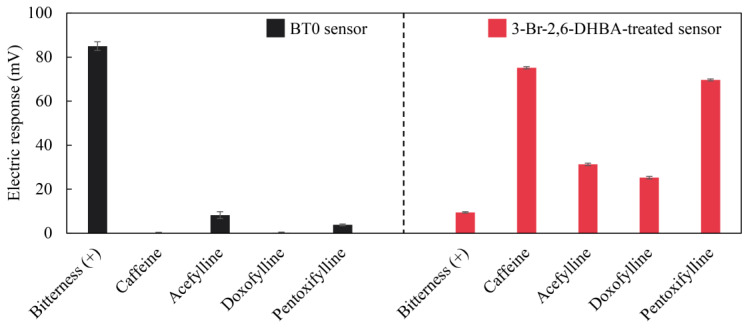
Responses of the BT0 sensor and the 3-Br-2,6-DHBA-treated sensor to quinine hydrochloride and non-charged bitter substances. The standard deviations (SD) of the outputs from both types of electrodes, BT0 (*n* = 6) and the 3-Br-2,6-DHBA-treated sensor (*n* = 12), were calculated separately and are shown by error bars.

As depicted in Figure 2, the BT0 sensor exhibited remarkable sensitivity to bitterness (+) sample, but it showed no response to caffeine and other non-charged pharmaceuticals with a xanthine scaffold. Conversely, the 3-Br-2,6-DHBA-treated sensor showed significant responses to non-charged bitter substances, including caffeine (75.12 mV), acefylline (31.29 mV), doxofylline (25.25 mV), and pentoxifylline (69.61 mV). The response to the caffeine sample was consistent with a previous paper [40]. These results show that the BT0 sensor had limitations in detecting certain non-charged bitter substances, such as caffeine and other pharmaceuticals. Conversely, these substances were detectable using the 3-Br-2,6-DHBA-treated sensor.

### 2.2. Taste Sensor Measurement and Sensory Tests of Acefylline and Doxofylline

#### 2.2.1. Concentration-Dependent Measurements Using 3-Br-2,6-DHBA-Treated Sensor and Sensory Tests

**Figure 3:**
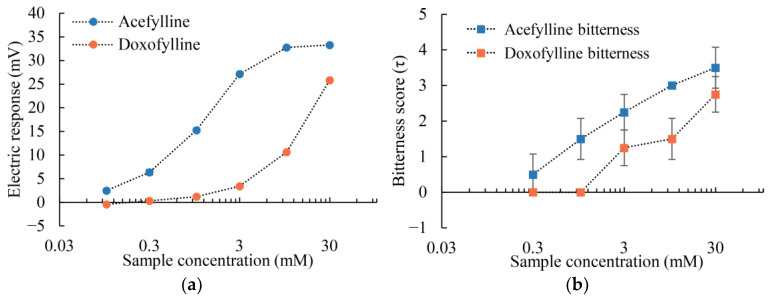
Concentration-dependent measurements of bitterness for acefylline and doxofylline: (**a**) Electric response obtained from the 3-Br-2,6-DHBA-treated sensor; (**b**) Sensory tests. Error bars represent the standard deviation (SD). Data in (**a**) represent *n* = 4 (electrode) × 3 (rotation) = 12 values, while data in (**b**) represent *n* = 4 values.

The results presented in Figure 3a show a positive correlation between the concentration of the sample solution and the sensor’s response, with the maximum responses in 30 mM sample solutions (acefylline = 33.27 mV and doxofylline = 25.83 mV). The sensor was more responsive to acefylline than doxofylline throughout the concentration range. Additionally, the relative standard deviation (RSD) values for acefylline remained consistently below 3% across the 1 to 30 mM the concentration range, as shown in Table 1. These results indicate that the 3-Br-2,6-DHBA-treated sensor is capable of detecting both acefylline and doxofylline, with superior sensitivity towards acefylline.

Sensory tests were conducted to evaluate the bitterness intensity for acefylline and doxofylline in sample solutions (0.3, 1, 3, 10, and 30 mM), with bitterness intensity scores (τ) assigned accordingly. As depicted in Figure 3b, there was a clear trend of increasing bitterness intensity with increasing sample concentration for both substances. At a concentration of 30 mM, acefylline exhibited the highest bitterness intensity score of 3.50 (τ), surpassing the bitterness intensity of doxofylline, recorded as 2.75 (τ). The outcomes of the sensory tests were found to correlate with the responses of the sensor, and this relationship is further explored in Section 2.2.2.

Certain substances with a xanthine scaffold, such as caffeine and etofylline, were detectable by the 3-Br-2,6-DHBA-treated sensor [30,39,40], operating through an allosteric mechanism discussed in the Introduction. Since acefylline possesses the same xanthine scaffold (as shown in Figure 1), the authors propose that the detection mechanism underlying the acefylline detection was also an allosteric one. When the 3-Br-2,6-DHBA-modified sensor electrodes were immersed in the acefylline sample solution, acefylline formed intermolecular H-bonds with the hydroxy group of 3-Br-2,6-DHBA. This intermolecular H-bonding can influence the formation of intramolecular H-bonding between the hydroxy and carboxyl groups in 3-Br-2,6-DHBA, thereby influencing the dissociation degree of carboxyl groups. Changes in the dissociation degree of these groups at the membrane site led to alterations in the surface charge density of the membrane, resulting in variations in the membrane potential. This mechanism is also applicable to doxofylline.

#### 2.2.2. Comparison with Taste Sensor Results and Sensory Test Outcomes

**Figure 4:**
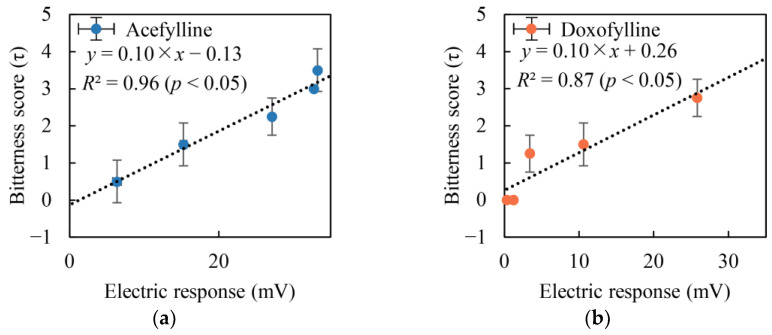
Relationships between electric response obtained from the 3-Br-2,6-DHBA-treated sensor and bitterness score in the human senses: (**a**) acefylline; (**b**) doxofylline. The standard deviations on the *x*- and *y*-axes represent differences between measurement errors (*n* = 12) and bitterness scores (*n* = 4), respectively.

In Figure 4a,b, a significant positive correlation is observed between the sensor responses and the results of sensory tests for acefylline and doxofylline in the concentration range of 0.3–30 mM, with corresponding *R*^2^ values of 0.96 and 0.87, respectively. These findings are in line with the Weber–Fechner law [53,54]. The data were provided in pairs, reflecting measurements from two distinct experiments at equivalent concentrations. Consequently, a paired *t*-test at a 95% confidence level was conducted for statistical analysis. For acefylline, the *p*-value was calculated to be less than 0.05. This result indicates that the difference between the sensor responses and the sensory test outcomes for acefylline was statistically significant. The statistical analysis yielded a *p*-value below 0.05 for doxofylline, demonstrating a significant difference between the sensor responses and the sensory test outcomes. Thus, employing the 3-Br-2,6-DHBA-treated sensor to assess the bitterness of acefylline or doxofylline was an effective approach. The actual analysis was performed using MATLAB software (version R2024a).

Both acefylline and doxofylline exhibit bronchodilation effects [49,50]. Additionally, acefylline is recognized as a potent pharmacological molecule for anti-cancer treatment [55], while doxofylline demonstrates better tolerability with lower dropout rates than theophylline [56]. Although sensory tests can evaluate these compounds, the error bars in the sensory test outcomes are significant (as shown in Figure 3b). Given the proportional relationship between electrical response and perceived bitterness intensity, using 3-Br-2,6-DHBA-modified taste sensors to mimic human perception of acefylline and doxofylline presents a practical and effective alternative.

### 2.3. Taste Sensor Measurement and Sensory Test for Allopurinol

**Figure 5:**
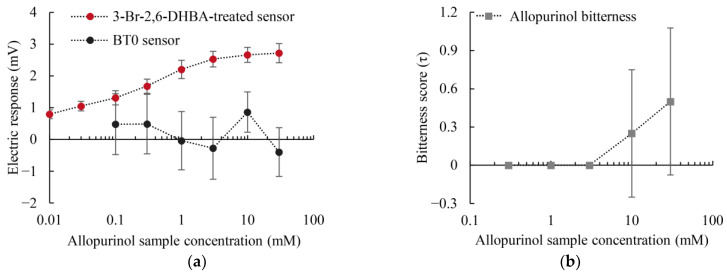
Concentration-dependent measurements of bitterness for allopurinol: (**a**) electric response obtained from the 3-Br-2,6-DHBA-treated sensor and BT0 sensor; (**b**) bitterness score results from sensory tests. Error bars represent the SD; data for the 3-Br-2,6-DHBA-treated sensor represent *n* = 8 (electrode) × 3 (rotation) = 24 values; data for the BT0 sensor represent *n* = 2 (electrode) × 3 (rotation) = 6 values; data for sensory tests represent *n* = 4 values.

The authors conducted concentration-dependent experiments for allopurinol using the BT0 sensor and 3-Br-2,6-DHBA-treated sensor. Figure 5a demonstrates that the response of the 3-Br-2,6-DHBA-treated sensor to allopurinol increased with the concentration of the allopurinol sample solution, reaching a maximum value of approximately 3 mV. The BT0 sensor did not show a clear dependence of response on concentration, exhibiting considerable variability and indicating poor repeatability. These results indicate that the BT0 sensor was unable to detect allopurinol. Given the low response of the 3-Br-2,6-DHBA-treated sensor to allopurinol, the authors inferred that allopurinol has relatively weak bitterness.

Figure 5b indicates that the maximum bitterness intensity of allopurinol was approximately 0.50 (τ) at 30 mM and approached 0.00 (τ) within the range of 0.3–3 mM. The data indicated that allopurinol was not detectably bitter at concentrations below a threshold of 3 mM, but became detectable at a relatively weak intensity at higher concentrations. Within the concentration range of 3 to 30 mM, the authors compared the sensory test outcomes with the sensor responses. The correlation coefficient (*R*^2^) reached 0.95, indicating consistency between sensor responses and sensory test outcomes. A paired *t*-test conducted on the taste sensor and sensory test data yielded a *p*-value below 0.05, indicating that the difference between the two sets of experimental data for allopurinol was statistically significant. These results confirmed the alignment between sensor-based assessments and sensory tests in evaluating the bitterness of allopurinol.

Allopurinol is not a xanthine derivative; it is a structural isomer of the natural purine base hypoxanthine, used to treat chronic gout [57]. Allopurinol is also utilized for preventing and treating oral mucositis (mouth ulcers) in cancer patients undergoing chemotherapy and radiation therapy [58]. For oral mucositis treatment, allopurinol needs to be applied directly to the oral mucosa or formulated into mouthwash solutions [59]. Clinically, allopurinol concentrations are often around 1 mg/mL, approximately 7.35 mM [60]. The sensor provided in this study was able to effectively detect allopurinol within the 0.01–30 mM concentration range, and demonstrated consistency with sensory test outcomes. Therefore, the 3-Br-2,6-DHBA-treated sensor is valuable and accurate in detecting allopurinol levels clinically.

### 2.4. Assessment of the Bitterness for Pentoxifylline

#### 2.4.1. Concentration-Dependent Measurements for Pentoxifylline Using 3-Br-2,6-DHBA-Treated Sensor

**Figure 6:**
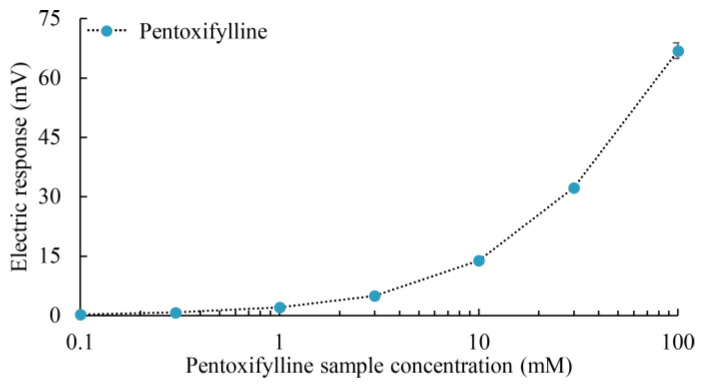
Electric response to pentoxifylline in sample solutions (0.1, 0.3, 1, 3, 10, 30, and 100 mM) measured using taste sensors with lipid/polymer membranes modified with 3-Br-2,6-DHBA. The mean values and SDs were calculated from 12 electrical response measurements (*n* = 4 electrodes × 3 rotations). Error bars represent the SDs.

In this section, the authors employed the 3-Br-2,6-DHBA-treated sensor to detect pentoxifylline. Four sensor electrodes, prepared as described in Section 3.2, were used for pentoxifylline measurements. As depicted in Figure 6, the sensor response increased proportionally with the sample concentration, reaching a maximum value of 66.89 mV with an RSD of 2.94%. This maximum response is comparable to that observed for caffeine, as shown in Figure 2, indicating that the sensor exhibited a remarkable response for pentoxifylline.

Regarding pentoxifylline, it is noteworthy that while it was withdrawn from the market in Japan due to re-evaluation as a cerebral circulation metabolism improver, it remains available in other countries as a vasodilator. Considering the withdrawal in Japan, the sensory tests for pentoxifylline were not included in this study.

Pentoxifylline, a xanthine derivative, possesses unique hemorheological properties. It is employed in treating various infectious, vascular, and inflammatory diseases in children, including the treatment of Kawasaki disease [61,62], necrotizing enterocolitis, and chronic lung disease in preterm neonates [51]. Clinically, adult patients typically receive 400 mg of pentoxifylline orally, approximately 1.44 mM, administered three times daily [63,64]. The 3-Br-2,6-DHBA-treated sensor detected 1 mM pentoxifylline with a response of 2.03 mV and an RSD of 11.87%; at 3 mM, the response was 4.99 mV with an RSD of 4.20%. This sensitivity is crucial, considering the wide range of applications for pentoxifylline in patient care.

#### 2.4.2. Assessment of the Bitterness for Pentoxifylline

The results from Figure 3b and Figure 5b indicate that the substances with a xanthine scaffold (i.e., acefylline and doxofylline) exhibited a stronger bitterness compared to allopurinol, thus emphasizing the significance of the xanthine scaffold in causing bitterness. The structural similarity in xanthine scaffolds between doxofylline and pentoxifylline, along with their comparable sensor responses (e.g., at 10 mM, doxofylline = 10.60 mV; pentoxifylline = 13.89 mV), suggests that the bitterness of pentoxifylline was similar to that of doxofylline. For instance, at 10 mM concentration, the bitterness intensity of doxofylline was measured at 1.50 (τ), suggesting the estimated bitterness for pentoxifylline to be approximately 1.50 (τ). Understanding the significance of the xanthine scaffold in causing bitterness provides valuable insights for molecular diversity researchers, enabling the design of new xanthine derivatives with enhanced pharmacological properties and reduced bitterness.

### 2.5. The Effectiveness of Xanthine Scaffold in Detection with 3-Br-2,6-DHBA-Treated Taste Sensors 

Previous studies [39,40] marked a significant advancement through employing 3-Br-2,6-DHBA-treated taste sensors to detect a wide range of xanthine derivatives. These include natural xanthine derivatives like caffeine, theobromine, and theophylline, as well as pharmaceuticals synthesized with xanthine scaffolds such as etofylline, proxyphylline, and diprophylline. Combined with this study and prior results, a total of eight xanthine derivatives have been detected. Therefore, the xanthine scaffold is a key factor for effective detection by the 3-Br-2,6-DHBA-treated sensor.

**Figure 7:**
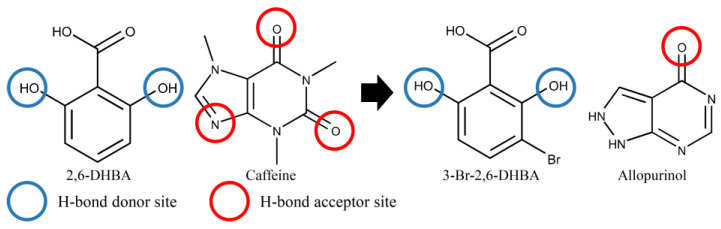
Distribution of H-bonding sites between 2,6-DHBA and caffeine, as well as 3-Br-2,6-DHBA and allopurinol.

Additionally, previous studies have investigated the molecular interactions between caffeine and various hydroxybenzoic acids (HBAs) using NOESY and ^1^H NMR experiments. One study indicated that the interaction between caffeine and 2,6-DHBA involves H-bonding between the hydroxy group of 2,6-DHBA and the carbonyl group or N(imidazole) of caffeine, along with π–π interactions between aromatic rings [31]. The similar structures of 3-Br-2,6-DHBA and 2,6-DHBA enable 3-Br-2,6-DHBA to mirror the H-bonding interaction between caffeine and 2,6-DHBA, thus forming H-bonding with caffeine at the hydroxy group of 3-Br-2,6-DHBA (Figure 7).

As for the detection of allopurinol by the 3-Br-2,6-DHBA-treated sensor, the diminished sensor response can be attributed to the absence of one oxygen in the carbonyl group (C=O) of allopurinol (Figure 7). This reduction in the number and probability of H-bonds between allopurinol and the 3-Br-2,6-DHBA-modified membrane resulted in the observed decline in sensor response. In future studies, molecules with purine scaffolds, such as mercaptopurine (lacking oxygen in the carbonyl group compared to xanthine), will be investigated. NMR experiments will further investigate the intermolecular interaction between 3-Br-2,6-DHBA and allopurinol.

## 3. Materials and Methods

### 3.1. Reagents

The lipid component in this study was tetradecylammonium bromide (TDAB). Dioctyl phenyl-phosphonate (DOPP) was used as the plasticizer. Polyvinyl chloride (PVC) was used as the supporting material. The DOPP, PVC, and TDAB were purchased from Dojindo Laboratories (Kumamoto, Japan), FUJIFILM Wako Pure Chemical Corporation (Osaka, Japan), and Sigma-Aldrich (St. Louis, MO, USA), respectively. Tetrahydrofuran (THF) was the chosen solvent for membrane production, and was also purchased from Sigma-Aldrich (St. Louis, MO, USA).

**Figure 8:**
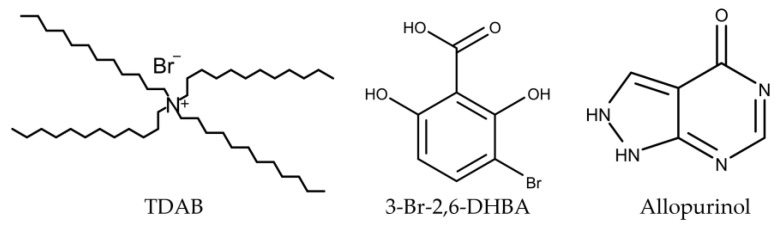
Structural formulas of xanthine, acefylline, doxofylline, pentoxifylline, TDAB, 3-Br-2,6-DHBA, and allopurinol.

Various substances were acquired for measurements, including caffeine, acefylline, doxofylline, pentoxifylline, and allopurinol. Acefylline was sourced from MedChemexpress (Monmouth Junction, NJ, USA), while doxofylline and pentoxifylline were provided by Tokyo Chemical Industry (Tokyo, Japan). Allopurinol was sourced from FUJIFILM Wako Pure Chemical Corporation (Osaka, Japan). Quinine hydrochloride was sourced from Kanto Chemical (Tokyo, Japan). The structural formulas of TDAB, 3-Br-2,6-DHBA, and allopurinol are shown in Figure 8.

Additionally, 0.5 mol/L potassium hydroxide (KOH) solution and 99.5% ethanol were used. The ethanol and KOH solution were purchased from Japan Synthetic Alcohol (Kawasaki, Japan) and FUJIFILM Wako Pure Chemical Corporation (Osaka, Japan), respectively. The 3-Br-2,6-DHBA was also obtained from FUJIFILM Wako Pure Chemical Corporation (Osaka, Japan).

### 3.2. Sensor Preparation: Lipid/Polymer Membrane and Surface Modification

As reported in previous studies [30,31], taste sensors with TDAB membranes have been utilized to detect non-charged bitter substances such as caffeine. The lipid membrane with TDAB synthesis involved mixing 10 mL of 3 mM TDAB in THF, 1.5 mL of DOPP, and 800 mg of PVC to achieve a homogeneous solution. This mixture was then poured into a petri dish, and THF was allowed to evaporate naturally at room temperature over a period of three days. A 3 mM TDAB membrane segment was securely attached to prepare the sensor electrodes.

Surface modification with 3-Br-2,6-DHBA enabled the sensor with the TDAB membrane to exhibit significant sensitivity for xanthine derivatives such as etofylline [40]. Thus, the sensor electrodes featuring TDAB membranes were soaked in a 0.03 wt% solution of 3-Br-2,6-DHBA for 72 h, facilitating the adsorption of 3-Br-2,6-DHBA onto the membrane’s surface. TDAB dissociated to carry a positive charge, while 3-Br-2,6-DHBA contains carboxyl groups, which can dissociate to carry a negative charge (Figure 8). Studies [65,66] have highlighted that liquid-membrane electrodes exhibit permselectivity towards ions of opposite charge, indicating that TDAB membranes exhibit electrostatic interaction with negatively charged ions, such as Br^−^ from TDAB and ionized 3-Br-2,6-DHBA. The hydrophobic characteristics of 3-Br-2,6-DHBA enabled its efficient adsorption onto the membrane’s surface.

### 3.3. Taste Sensor Measurement

#### 3.3.1. Measurement Procedure of Taste Sensor

**Figure 9:**
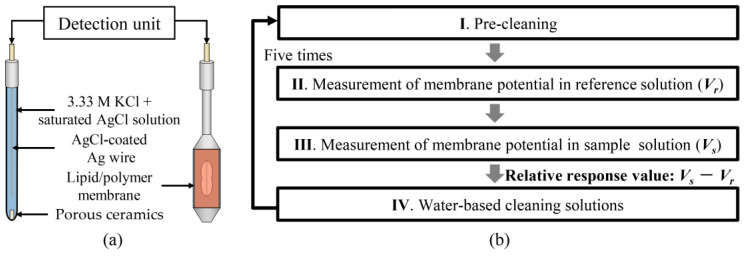
(**a**) Structure of the sensor and reference electrodes. (**b**) The measurement process of the taste sensor.

All taste measurements in this study were based on the commercialized taste sensing system (TS–5000Z, Intelligent Sensor Technology, Inc., Kanagawa, Japan). A reference electrode with AgCl-coated Ag wire and the sensor electrode with TDAB membrane were connected to a detection unit (Figure 9a). The inner solution for the sensor and reference electrodes was a mixture of 3.33 M KCl and saturated AgCl. The measurement procedure of the taste sensor is shown in Figure 9b. Following a pre-cleaning step, the detection unit was immersed in the reference solution, resembling human saliva and possessing little taste [67]. Next, the sensor and the reference electrodes were placed into a sample solution to obtain *V**_s_***. The difference between *V**_s_*** and *V**_r_*** was calculated as the relative response value. Finally, the membrane surface underwent cleaning with a water-based cleaning solution composed of 10 mM KOH, 100 mM KCl, and 30% ethanol by volume. The taste sensor measurement procedure was repeated consecutively five times intra-day, with data from the third to fifth iterations utilized for subsequent analysis.

#### 3.3.2. Selectivity Measurement for Bitter Substances Using BT0 Sensor and 3-Br-2,6-DHBA-Treated Sensor

A previous study [28] has demonstrated the BT0 sensor’s ability to detect the bitterness of pharmaceuticals, including quinine hydrochloride and other strongly hydrophobic drugs. In this section, the authors aimed to determine the sensitivity of the BT0 sensor to non-charged bitter substances such as caffeine, acefylline, doxofylline, and pentoxifylline. The concentrations of the tested sample solutions are shown in Table 2, where the concentrations of caffeine and quinine hydrochloride were consistent with those employed in the previous study [40]. Positively charged and non-charged bitter substances were dissolved in the reference solution for sample preparation, respectively.

Two BT0 sensors were obtained from Intelligent Sensor Technology (Kanagawa, Japan), and four sensor electrodes with TDAB membranes were prepared using surface modification with a 0.03 wt% 3-Br-2,6-DHBA solution. Each experiment was conducted five times consecutively, and the data from the third to fifth times were taken for analysis.

#### 3.3.3. Measurement for Acefylline, Doxofylline, and Pentoxifylline Using 3-Br-2,6-DHBA-Treated Sensor

Concentration-dependent measurements were conducted to verify the sensitivity of the 3-Br-2,6-DHBA-treated sensor to acefylline, doxofylline, and pentoxifylline. Due to their limited solubility, the concentrations of acefylline and doxofylline were set at 0.1, 0.3, 1, 3, 10, and 30 mM in the reference solution. For pentoxifylline, the sample concentrations were set at 0.1, 0.3, 1, 3, 10, 30, and 100 mM in the reference solution.

Four sensor electrodes were prepared using the method described in Section 3.2. Each experiment was repeated five times consecutively within a single day, and the data from the third to fifth trials were used for analysis. Mean values and SDs were calculated based on 12 electrical response values obtained from n = 4 electrodes × 3 rotations.

#### 3.3.4. Measurement for Allopurinol Using BT0 Sensor and 3-Br-2,6-DHBA-Treated Sensor

A previous study [27] found no concentration-dependent response when using bitterness sensors like BT0 to detect allopurinol within the 0.01 to 0.1 mM concentration range. In this section, the authors aimed to verify whether the BT0 sensor exhibited concentration-dependent responsiveness to allopurinol at higher concentrations (0.1, 0.3, 1, 3, 10, and 30 mM). Additionally, to further demonstrate the detection capability of the 3-Br-2,6-DHBA-treated sensor for allopurinol, we expanded the range of allopurinol sample concentrations to include 0.01, 0.03, 0.1, 0.3, 1, 3, 10, and 30 mM. Eight 3-Br-2,6-DHBA-treated sensor electrodes were prepared for allopurinol detection using the method described in Section 3.2.

### 3.4. Sensory Tests for Acefylline, Doxofylline, and Allopurinol

A panel of four well-trained members (one healthy male and three females with an average age of 34.75 ± 20.84 years) who were able to discriminate bitterness in each standard quinine hydrochloride solution sample conducted sensory evaluation of the test samples (acefylline, doxofylline, and allopurinol) according to the procedure outlined in a previous study [68]. Quinine hydrochloride served as the bitter standard sample, and the bitterness scores (τ) were defined as 0, 1, 2, 3, and 4 for concentrations of 0.01, 0.03, 0.10, 0.30, and 1.00 mM, respectively. One week before and one hour before the actual start of the sensory test, panel members were shown the protocol of the sensory test, and also received detailed explanations regarding the information about the test samples and standard solutions using written materials.

Sensory panelists began by tasting quinine hydrochloride solutions at specified concentrations and memorizing the taste associated with each bitterness score. They held 2 mL of the quinine hydrochloride solutions in their mouth for 5 s and recalled the bitterness scores. Subsequently, panelists evaluated the bitterness of the test samples using predetermined scores. They held each sample in their mouth for 5 s and provided corresponding bitterness scores. After the 5 s period, panelists immediately expelled the test sample from their mouths, followed by thorough gargling. A 20 min break was given between each sample test, including the quinine hydrochloride standard samples. The Ethical Committees of Mukogawa Women’s University granted pre-approval for the design of the sensory test (No. 23–94) in advance, on 16 December 2023.

## 4. Conclusions

Prior work has documented the effectiveness of utilizing a taste sensor with an allosteric approach in detecting non-charged pharmaceuticals, including etofylline, proxyphylline, and diprophylline, which share a xanthine scaffold. However, this method has not been extensively applied to other non-charged pharmaceuticals with a xanthine scaffold, including acefylline. In this study, the researchers utilized a taste sensor equipped with TDAB membranes modified by 3-Br-2,6-DHBA, in conjunction with sensory tests, to assess bitterness in substances with a xanthine scaffold, specifically acefylline and doxofylline, as well as allopurinol. The sensor demonstrated reliable sensitivity towards these substances. The samples with a xanthine scaffold showed higher sensor responses and a stronger bitterness intensity, whereas allopurinol exhibited lower sensor responses and relatively weaker bitterness. Furthermore, the researchers used the 3-Br-2,6-DHBA-treated sensor to evaluate the bitterness of pentoxifylline, suggesting it was stronger than allopurinol due to its xanthine scaffold. This study indicates that the 3-Br-2,6-DHBA-modified taste sensor is a valuable complementary tool for assessing the bitterness of substances with a xanthine scaffold. Most notably, this study expands the assessment range of the taste sensor from xanthine derivatives to hypoxanthine analogues (i.e., allopurinol). Nevertheless, many non-charged pharmaceuticals without a xanthine scaffold remain to be tested, such as mercaptopurine, which is used to treat leukemia and has a purine structure. Future work should include follow-up studies to evaluate the bitterness of mercaptopurine and additional non-charged pharmaceuticals.

## Figures and Tables

**Table 1:** Repeatability (intra-day) RSD of the 3-Br-2,6-DHBA-treated taste sensor to acefylline and doxofylline.

Concentration (mM)	Repeatability (Intra-Day) RSD [%]	
	Acefylline	Doxofylline
0.3	9.15	35.82
1	2.87	16.18
3	0.61	6.02
10	0.81	1.92
30	0.81	0.78

**Table 2:** Components and concentrations of samples.

Sample	Composition	Concentration
Bitterness (+)	quinine hydrochloride	0.1 mM
Non-charged bitter substances	caffeine	100 mM
	acefylline	30 mM
	doxofylline	30 mM
	pentoxifylline	100 mM

## Data Availability

The data presented in this study are available on request from the corresponding author.
